# Protocol of the MOVI-ageing randomized controlled trial: a home-based e-Health intervention of cognitively demanding exercise for the improvement of cardiorespiratory fitness and cognitive function in older individuals

**DOI:** 10.3389/fpubh.2023.1298316

**Published:** 2023-12-22

**Authors:** Celia Alvarez-Bueno, Maribel Lucerón-Lucas-Torres, Abel Ruiz-Hermosa, Irene Sequí-Dominguez, Luis Carlos Venegas-Sanabria, Maria Medrano-Echeverria, María Eugenia Visier-Alfonso, Beatriz Rodriguez-Martin

**Affiliations:** ^1^Health and Social Research Center, Universidad de Castilla-La Mancha, Cuenca, Spain; ^2^Universidad Politécnica y Artística del Paraguay, Asunción, Paraguay; ^3^Universidad de Extremadura, ACAFYDE Research Group, Cáceres, Spain; ^4^Escuela de Medicina y Ciencias de la Salud, Universidad del Rosario, Bogotá, Colombia; ^5^Hospital Universitario Mayor – Méderi, Bogotá, Colombia; ^6^Research Institute for Innovation and Sustainable Food Chain Development (IS-FOOD), Department of Health Sciences, Public University of Navarre, Pamplona, Spain; ^7^IdiSNA, Navarra Institute for Health Research, Pamplona, Spain; ^8^Centro de Investigación Biomédica en Red Fisiopatología de la Obesidad y Nutrición (CIBERobn), Instituto de Salud Carlos III, Madrid, Spain; ^9^Department of Nursing, Physiotherapy and Occupational Therapy, University of Castilla-La Mancha, Talavera de la Reina, Spain

**Keywords:** ageing, clinical trial, epidemiology, patient-centered care, primary prevention, sports medicine

## Abstract

**Objective:**

To describe the protocol of the MOVI-ageing randomized controlled trial, a home-based eHealth intervention of cognitive-demanding exercise for older adults, in improving global cognitive function and basic cognitive functions, cardiorespiratory fitness, and muscle fitness.

**Methods:**

This randomized controlled trial will include participants identified in the social centers of Cuenca and Talavera de la Reina who agree to participate and provide informed consent. Adults aged 60–80 years of both genders retired regardless of the reason for retirement, who do not meet frailty criteria according to Fried criteria, and without cognitive impairment will be invited to participate. This study will be developed in two phases: (i) a 12-week randomized efficacy/feasibility trial and (ii) a large-scale implementation randomized trial phase with a 12-week follow-up following similar procedures. In addition, a qualitative study on barriers to and facilitators of the implementation of the physical exercise intervention using eHealth for older people will be conducted. Participants will have access to a platform including videos of cognitively demanding physical exercise. The participants will be remotely and off-line guided through the physical exercise intervention, and the research team will be able to check the degree of compliance with the program and its correct execution. The participants will receive feedback on their compliance with the routines and reinforcement messages.

**Implications:**

The implementations of the findings and their inclusion in guidelines may directly impact in older people’s life, and relatives, through the prevention of morbidity and the reduction of years lost to disability. These benefits may be reflected in the reduction of economic expenditure by reducing the demand for social and health care services.

**Ethics:**

The Clinical Research Ethics Committee of the ‘Virgen de la Luz’ Hospital in Cuenca approved the study protocol (registration number: 2022/PI3222). In addition, this protocol was previously registered in Clinicaltrials.gov (Number: NCT05928078).

## Introduction

Although there is significant heterogeneity in the aging process, it is accompanied by a general decline in functional capacity, mainly related to the process of sarcopenia and to oxidative and inflammatory processes associated with arterial aging (1, 2). Age-related physiological and pathological changes and the complications arising from them have a major impact on lifestyle (1). Good muscle capacity is essential for the maintenance of functional ability and the promotion of quality of life (QoL). Aging-related declines in strength and respiratory capacity are well-known causes of decreased walking speed and increased mobility limitations and disabilities among older individuals, all of which are risk factors for institutionalization and mortality (3). In addition, declining health is associated with functional limitations, restricted social interaction, and impaired mental health status, including reduced cognitive function, neurodegeneration, and the development of dementia (1).

Moreover, cognitive impairment is one of the most common causes of disability in developed countries among the older adults (4). Cognitive decline and dementia place social and economic pressure on the governments of nations, which is expected to increase as a new case of dementia is diagnosed every 4 s (5–7). This means that approximately 115 million older people worldwide are likely to suffer from Alzheimer’s disease/dementia by 2050. Although the economic costs are enormous, the combined economic and social burden of dementia is even more daunting, as it corresponds to the burden of people with dementia and their families and is, therefore, difficult to calculate (8). In view of this situation, the World Health Organization (WHO) emphasizes the importance of taking comprehensive actions against cognitive impairment and dementia and urges governments and institutions to develop prevention strategies that reduce the risk of cognitive decline (6, 9).

Without ignoring the importance of nutritional and lifestyle modification interventions, physical exercise is the primary therapeutic strategy for the prevention of disease and associated decline in functional capacity (10). Evidence suggests that enriching physical exercise interventions with a cognitive challenge could maximize the neuroplastic properties of physical exercise. The additive effects of cognitively demanding physical exercise could be explained by the temporal nature of peripheral brain-derived neurotrophic factor (BDNF), which returns to basal levels 10–60 min after physical exercise, requiring a temporal succession of cognitive demands to optimally benefit from these neurotrophic effects (11). Furthermore, the brain plasticity favored by increasing cell proliferation and synaptic plasticity is “guided” by cognitive training by increasing the number of new-born neurons that survive and their integration in new neural networks, as well as the increased number of synapses in preexisting neural networks (12). It has been suggested that these effects could be observed regardless of age, and therefore cognitive decline could be preventable (13).

## Aims

The purpose of this paper is to describe the protocol of the MOVI-ageing randomized controlled trial, a home-based eHealth intervention of cognitive-demanding exercise for older adults, in improving global cognitive function and basic cognitive functions, cardiorespiratory fitness, and muscle fitness.

## Methods

### Design

For the design of this protocol, the CONSORT guidelines for randomized controlled trials studies (14) and the Consolidated Criteria for Reporting Qualitative Studies (COREQ) (15) were used. The MOVI-ageing randomized controlled trial will include a mixed design, following quantitative and qualitative approaches.

The quantitative study will be developed in two phases: (i) an efficacy and feasibility study and (ii) an implementation study. The former will be aimed at testing the efficacy of the MOVI-ageing intervention in improving key outcome variables, refining the intervention, and examining potential biases of the intervention. During this phase, a qualitative approach will be used as feedback to define the development of the platform, as evidence indicates that when users are involved in the design and evaluation of the feasibility study, the efficacy of the implementation increases. The latest will be developed following the same methodological procedures to test the MOVI-ageing intervention in a larger sample size.

### Recruitment and allocation

The Clinical Research Ethics Committee of the ‘Virgen de la Luz’ Hospital in Cuenca has approved the study protocol (registration number: 2022/PI3222). The development of this project will be supported by the Health Care Services of Cuenca. In addition, this protocol has been previously registered in Clinicaltrials.gov (Number: NCT05928078).

Potential participants will be accessed through key informants in the social centers of Cuenca and Talavera de la Reina. The research team will contact the potential participants by phone to offer them participation in the study. In addition, flyers and posters will be located in places where older people could potentially develop leisure activities (including but not limited to libraries and older adults’ centers) with the contact information of the research team.

**Figure 1:**
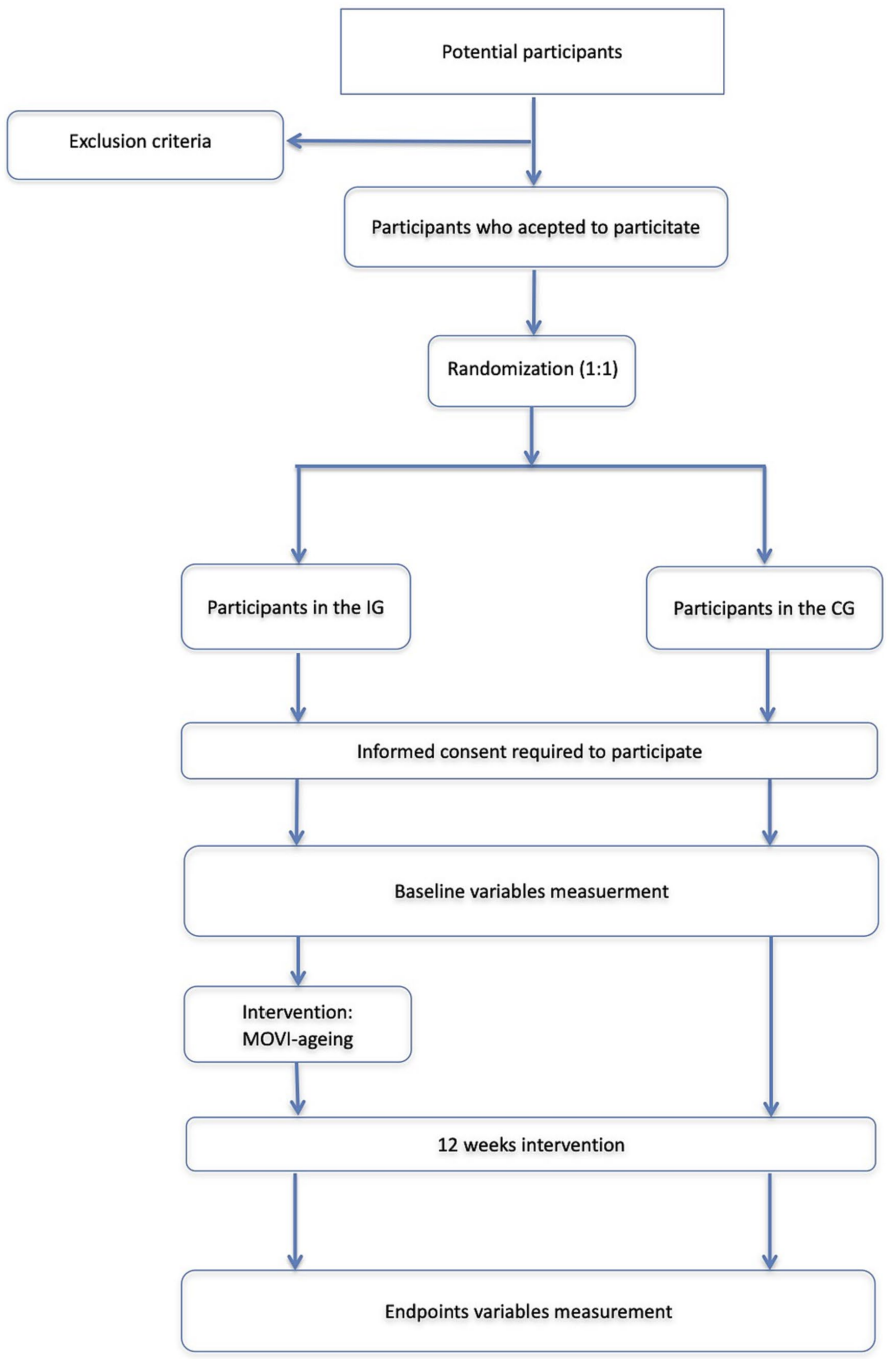
Flow chart of trial participants. CG, control group; IG, intervention group.

During the information process of the participants, the research team will inform them that they can drop out of the study as they request. After agreement to participate and informed consent, participants will be randomly distributed (1:1) to the intervention (IG) or control group (CG) using EPIDAT 4.2 software (Figure 1).

Although the application of a physical intervention program cannot be blinded to participants, the investigators involved in measurements will be blinded to the group to which the participants belong. With the same aim, each participant will receive an identification number to blind his/her allocation during the analysis of data. The identification number and the personal data of the participants will be stored in different data bases and will be crossed only in case to detect anormal values during the measurement processes.

### Participants: inclusion and exclusion criteria

Participants will be invited to participate when meeting the following inclusion and exclusion criteria.

Inclusion criteria. Individuals: (1) aged 60–80 years of both genders; (2) retired regardless of the reason for retirement; (3) not meeting frailty criteria according to Fried criteria; (4) are without disabilities for activities of daily living; (5) live independently; (6) without cognitive impairment as determined by the Mini-Mental test greater than 24 (considering the degree of schooling of the participants); and (7) able to walk at least 20 meters with or without walking aids.

Exclusion criteria. Individuals will be excluded when they have any of the following: (1) mobility problems; (2) serious health problems (e.g., recent myocardial infarction, uncontrolled diabetes, or uncontrolled hypertension); (3) orthopedic or neurological disease that prevents training; (4) Alzheimer’s disease or dementia; (5) progressive or terminal illness; (6) acute or chronic illness; (7) history of heart attack; (8) history of vertigo or recent head injury; (9) health problems that may affect the ability to perform physical exercise (e.g., acute and painful joint inflammation, impaired joint function, acute and painful joint inflammation, or deterioration in the ability to perform physical exercise); (10) intake of medications that act at the neuron level (e.g., psychotropic medications); (11) signs of incipient depression; or (12) pathology that impedes the use of the computer application through which the physical exercise program will be developed.

### MOVI-ageing intervention: description

The MOVI-ageing intervention will be developed as a randomized controlled trial with two arms in which participants will be randomly assigned to the IG, in which a home-based e-Health intervention of cognitively demanding exercise will be performed, or to the CG in which participants will continue their regular daily activity.

The e-Health intervention will be delivered using a platform including videos that will guide the participants in the realization of the movement during the cognitively demanding physical exercises. The participants will follow the videos remotely and off-line, and the users will have to imitate the movements of the trainer. Through the platform, it will be possible to evaluate the degree of compliance with the exercise program and the correct performance, as the system will accurately track and measure the participant’s limb movement through the camera and provide the research team with an objective measure of the patient’s movements and progress. Research team will be able to check this information during the development of the session or at any time of the clinical trial duration.

**Figure 2:**
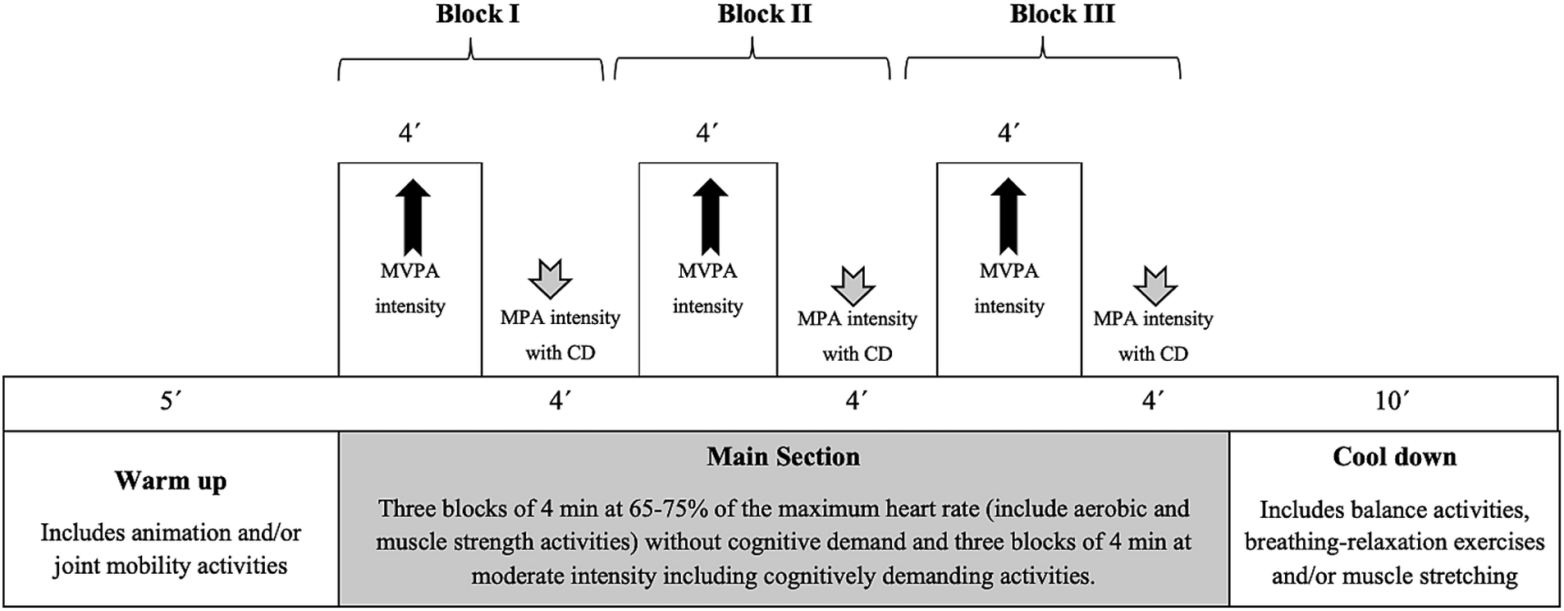
Basic design of a MOVI-ageing session. MVPA, Moderate-vigorous physical activity; MPA, moderate physical activity; CD, cognitive demands.

The recorded videos, designed by a team of experts including nurses, psychologists, physical activity graduates, and geriatricians, will include cognitively demanding exercises involving both basic cognitive functions (i.e., inhibition, working memory, and cognitive flexibility) and higher cognitive functions (i.e., spatial orientation, numerical calculation, and semantics). Each video will include a standardized physical exercise program structured as follows: 5 min warm-up (including animation and/or joint mobility activities), three blocks of 4 min at 65–75% of the maximum heart rate (including aerobic and muscle strength activities) without cognitive demand, three blocks of 4 min at moderate intensity including cognitively demanding activities, and 10 min of cool-down (including balance activities, breathing-relaxation exercises, and/or muscle stretching) (Figure 2).

To ensure the standardization of the program, at the beginning of the intervention, participants in the IG will attend a 2-h training session for the use of the platform and will be instructed in the control of their heart rate during the execution of the physical exercise program to be able to control the intensity of the physical exercise they perform. Users will be provided with indications to carry out the activities and will be required to perform at least 3 sessions per week of cognitively demanding physical exercise through the platform for 12 weeks.

#### Criteria for nonadherence and prolongation of training

Compliance with the exercise program will be regularly monitored through the intervention. To be considered in the analyses, participants must attend at least 70% of the sessions. If, due to unexpected circumstances, a participant does not comply with the exercise program within the 12 weeks, the training program may be extended for up to 4 weeks. Finally, in case of unexpected adverse effects of the intervention, the participant will be clinically followed-up, and the research team will record the reasons for drop out.

#### MOVI-ageing: monitoring and adherence

To improve adherence to the intervention, a telephone number and an email address will be available for participants for any doubt or information they need. Weekly information on the amount of physical activity performed will be emailed to the participants. The research team will contact participants monthly to check their satisfaction with the intervention.

### Variables

Measurement of the variables will be performed on all participants at the beginning and at the end of each phase of the study. Each participant will be scheduled at the facilities of the research team to carry out the measurements of all variables on a single day, and investigators will be previously trained to standardize the measurements. The variables will be measured following standardized and validated processes with the following tools (Table 1):

1. Basic executive functions: using the NIHtool (16) and including (a) Attention using the Eriksen flanker Task test where a 40-trial block will be presented consisting of a pseudorandom sequence of congruent and incongruent trials. Scoring will be calculated based on accuracy and reaction time; (b) Working memory using an adaptation of the Mungas List Sorting task where a series of illustrated pictures will be presented visually and orally. Participants will be asked to verbally repeat the names of the pictures in order of size, from smallest to largest. Two list versions will be presented, the first presenting items in one category (i.e., animals), and the second presenting items in 2 categories (i.e., animals and food) in which participants will be asked to organize items by category and size; and (c) cognitive flexibility using the DCCS (Dimension Change Card Sort) test. This tool will present a 30-trial block of mixed by “color” or “shape” stimulus, where participants will be asked to adapt their response according to the relevant dimension.
2. Global cognitive function with the Mini-Mental State Examination (MMSE) (17), which consists of 30 items grouped into 11 categories (including visual space, memory, naming, attention, calculation, abstract, orientation, and language function), with higher scores representing better cognitive functioning. The MMSE has been widely used in older people and has excellent reliability and validity (18). In this study, cognitive impairment will be defined as an MMSE score of less than 24 for respondents with 8 years of education or more and less than 20 for those with less than 8 years of education (19).
3. Health-related quality of life (HRQoL) with the SF-12 test (20), which aimed to evaluate the intensity and/or frequency of people’s state of health. The scale is composed of 12 items that could be answered by a Likert-type scale. This questionnaire provides information on eight subscales: physical functioning, physical role, bodily pain, general health, vitality, social functioning, emotional role, and mental health. These eight subscales comprise the physical and mental domains of patients’ HRQoL, where the higher the score is, the better the HRQoL. The SF-12 is a valid and reliable instrument.
4. Depression, anxiety, and stress will be assessed with Yesavage’s 15-item Geriatric Depression Scale (GDS) questionnaire (21, 22), which includes questions related to affect, in-activity, irritability, isolation, distressing thoughts, or negative judgments. The questions have dichotomous answers (yes/no) regarding the last week.
5. Cardiorespiratory fitness measured with the 6-min walking test (6MWT), where participants will be instructed to “walk as far as possible” for 6 min back and forth along a corridor 100 feet in length (23). Participants will be encouraged to walk but permitted to slow down, stop, and rest when needed. This test reflects the overall functional ability and the impact of multiple factors on exercise capacity (24).
6. Muscular strength will be measured by upper extremity dynamometry with the digital TKK 5401 Grip-D (Takey^®^, Tokyo, Japan). This test measures the upper body strength in kilograms. The test will be performed twice with the right hand and twice with the left hand; the mean average of the 4 measurements will be calculated.
7. Physical activity will be measured with the Axivity 6-axes accelerometers (Axivity LTd.) for seven consecutive days (including nights), with a fixed frequency of 30.0 Hz to collect raw acceleration data measured in “g” for each movement axis (x, y, and z). Data will be expressed in units of milligrams (1,000 mg = 1 g = 9.81 m/s^2^). We will consider as valid registers of ≥5 days, including 1 weekend day.
8. Physical function will be measured with the Short Portable Physical Battery scale (SPPB), (25) which measures balance, walking speed, and standing up and sitting on a chair 5 times. During the balance test, the participants will be required to maintain 3 positions for 10 s each: feet together, semitandem, and tandem. In the walking speed test, the participant will be encouraged to walk a 4 m distance at his/her usual pace. This test will be performed 2 times, and the shortest time will be recorded. Finally, the total time the participant spent standing up and sitting 5 times on a chair will be recorded. Each test could be scored from 0 (worst performance) to 4 (best performance). In addition, the score for the total battery will be the sum of the 3 tests and ranges from 0 to 12.
9. The concern of falling with the Falls Efficacy Scale International (FES-I) (26), which includes the possibility of falling when performing 16 different everyday life activities. Each item could be classified as follows: 1 = not at all concerned, 2 = somewhat concerned, 3 = fairly concerned, and 4 = very concerned. The total score could range from 16 to 64, with lower scores indicating a low fear of falling.
10. Anthropometric variables, including (i) Weight measured with a Seca^®^ 861 scale, with the participant barefoot and lightly dressed; (ii) Height: measured with a Seca^®^ 222 wall-mounted stadiometer, with the participant barefoot and in an upright position while the sagittal midline of the back touches the vertical bar; and (iii) Body mass index (BMI) calculated using the mean of the two measurements of weight and height as follows: weight (kg)/height^2^ (m^2^).
11. Body composition, including (i) waist circumference reported as the mean of three measurements using a flexible tape measure at the midpoint between the last rib and the iliac crest at the end of a normal exhalation. (ii) Body fat percentage reported as the mean of two measurements using an eight-electrode Tanita^®^ Segmental-418 bioimpedance system (Tanita Corp. Tokyo, Japan). And (iii) Body composition by densitometry (DXA), which allows us to determine fat mass, lean mass, bone density, and bone mineral content.
12. Blood pressure measured using an OMRON^®^ HEM-907 and reported as the mean of two measurements 5 min apart and after a 5-min rest period.
13. Biochemical determinations, including (1) Fasting plasma glucose, apolipoproteins A1 and B, insulin ultrasensitive protein C and endothelial glycocalyx (syndecan-1, syndecan-4 and heparan sulfate proteins) with the Abbott^®^ Cobas 8000 Roche Diagnostics^®^ system; and (2) HbA1c, determined by HPLC (high-performance liquid chromatography) using the ADAMS A1c HA-8180V analyser (Menarini Diagnostics^®^), a method certified by the NGSP (National Glycohemoglobin Standardization Program) and the IFCC (International Federation of Clinical Chemistry and Laboratory Medicine). In addition, BDNF will be measured by enzyme-linked immunosorbent assay (ELISA) (R & D Systems, Minneapolis, MN, USA).
14. Subclinical markers of atherosclerosis and vascular function, including (1) pulse wave velocity (PWv), radial augmentation rate (rAIx), and central augmentation rate (cAIx), which will be measured with the SphygmoCor system (AtCor Medical Pty Ltd. Head Office, West Ryde, Australia); (2) endothelial function: with the ENDO-PAT; and (3) 24-h blood pressure with the MAPA Mobil-O-graph.
15. Adherence to the Mediterranean diet will be measured with the Mediterranean Diet Adherence Screener (MEDAS) questionnaire (27), which includes 12 questions on food consumption frequency and 2 questions on food intake habits considered characteristic of the Spanish Mediterranean diet. Each question will be scored 0 or 1, where the final score could range from 0 to 14.
16. Sleep habits and quality of sleep will be measured with the Pittsburgh Sleep Quality Index (PSQI) (28), which consists of 24 questions, including 19 self-reported and 5 that require secondary feedback from a room or bed partner. Only the self-reported items will be used for the quantitative evaluation of sleep quality, with scores ranging from 0 to 21.
17. Comorbidities measured with the Charlson comorbidity index (29), which consists of the register of 17 comorbidities, with two subcategories for diabetes and liver disease. The total score will be summed from the weighted total mortality risk and disease severity.
18. Level of education: measured as the highest level of education achieved by each participant, classified as: cannot read or write, no education, incomplete primary education, primary education, high school graduation, high school graduation, intermediate university studies, or higher university studies.

**Table 1:** Study variables.

Type of variable	Specific variables
Primary endpoints	Executive function: inhibition, working memory and cognitive flexibility
	Global cognitive function
Secondary endpoints	Health-related quality of life
	Depression, anxiety, and stress
	Cardiorespiratory fitness
	Muscle strength
Other endpoints	Physical activity: accelerometry
	Physical function
	Concern of falling
	Anthropometry: weight, height, BMI
	Body composition: waist circumference, body fat, body composition
	Blood pressure:
	Blood tests: Fasting plasma glucose, apolipoproteins A1 and B, insulin ultrasensitive protein C, HbA1c, and BDNF
	Markers of atherosclerosis: PWv, rAIx, cAIx, endothelial function, and 24-h blood pressure
	Food consumption
	Sleep habits and quality of sleep
	Comorbidities
Possible confounding factors	Age
	Sex
	Level of education

BMI, body mass index; cAIx, central augmentation rate; HbA1c, hemoglobin glucose; PWv, pulse wave velocity; rAIx, radial augmentation rate.

It should be considered that measurement of accelerometry, body composition with DXA, biochemical determinations, and subclinical markers of atherosclerosis will only be performed in the efficacy and feasibility study.

### Statistical analysis

#### Sample size

Previous studies (30) have shown that a combined program of exercise and cognitive training for 3 months can improve working memory in cognitively healthy older adults, with an approximate effect size of 0.9. Considering the characteristics of our study and using GPower software, we calculated that a sample size of 21 participants per group would be needed to achieve a power of 0.8, with an alpha of 0.05 and a beta of 0.2. Assuming a dropout rate of 15%, a final sample size of 24 participants per group would be required for the efficacy and feasibility study. The sample size for the implementation study will be calculated based on the results of the previous phase.

#### Analyses of outcomes

After measurements, participants’ data will be entered into a database by two independent researchers. Blinding on handling data will be assured by separating measurement values from general participant information.

The statistical analysis plan can be summarized as follows: (i) after checking that randomization has been effective, we will winsorize the variables to limit the influence of extreme values; this will be done by replacing values below the 1st percentile with the 1st percentile value and those above the 99th percentile with the 99th percentile value. (ii) Mixed regression models will be used in which each outcome variable will be the dependent variable, interventions will be treated as fixed effects (1 = IG and 0 = CG), and the models will be adjusted for baseline reference values and age. The results will be expressed as absolute differences in changes in the variables between baseline and final measurement. When the dependent variable is dichotomous, odds ratios (ORs) will be calculated. Finally, (iii) analyses will be conducted from an intention-to-treat perspective, whereby participants will remain in the group to which they were originally assigned, regardless of the degree of compliance with the intervention.

The results will be considered statistically significant when *p* < 0.05. The analyses will be performed using STATA16.

### Qualitative approach

A qualitative study will be carried out following Giorgi’s phenomenological approach with the aim of describing the meanings of phenomena from participants’ experiences (31, 32). The information obtained from the qualitative study on barriers to and facilitators of the implementation of the cognitively demanding physical exercise intervention using eHealth for older people will be used to adapt the MOVI-ageing application.

Focus groups will be used because of their capacity to generate in-depth information on the perceptions and opinions of the phenomenon through the interaction of the participants and to explore a wide variety of opinions on the subject (33). Giorgi’s descriptive phenomenology approach will be used to explore the barriers and facilitators of older people’s use of a computer application for physical exercise, information that will guide the development and implementation of the MOVI-ageing intervention (31). As a data collection technique, focus groups will be conducted in a purposive sample of homogeneous groups of older people of both genders (men and women), socioeconomic level (low, medium, high), educational level (university or intermediate level studies, school graduate, no studies) and physical condition (assessed with the SF-12 scale). The criteria of intragroup homogeneity and intergroup heterogeneity will be followed to ensure that participants can express themselves freely.

The focus groups will be conducted by experts in qualitative methodology who will share a common protocol that will include the methodology of data collection and the script of topics. Each focus group will have a moderator, who will have the script of themes, conduct the focus group, and launch the questions, and another researcher who will act as an observer. The focus groups will be conducted in a neutral, comfortable, quiet, and private place, will last between 60 and 120 min, will be audio-recorded after obtaining the participants’ permission, and will include between 5 and 8 participants per group. At least two focus groups will be conducted, but the final number will be conditioned by the data saturation criterion to be reached when no novel analytic information is obtained (34).

Data collection and analysis will follow an interactive circular process so that the data collected in each focus group will serve to refine the topic script of the following groups. In addition, all data will be compared to each other through the constant comparison method. As data verification strategies, the focus groups will be audio-recorded and transcribed verbatim and subsequently anonymized for analysis. In addition, the interview transcripts will be returned to the participants for their agreement with the interviews. During the analysis phase, data will be grouped into themes and subthemes following the following steps of Giorgi’s phenomenological approach: (1) collect and describe phenomenological data, (2) read the full description in the transcribed texts, (3) break descriptions into meaning units that are as descriptive as possible, avoiding precocious interpretation of the results, (4) group the units by common meanings, forming clusters of meanings, and (5) interpret the clusters of meanings and identify the themes that will show the meaning of the phenomenon (31). Two researchers with expertise in qualitative methodology will independently perform the data analysis, subsequently agreeing on the results; in case of disagreement, a third researcher will mediate. The triangulation of data by three researchers will allow the emergence of different perspectives and deepen the analysis, increasing the validity of the findings. Atlas-ti 9.0 software will be used as an aid during this phase. The credibility, transferability, reliability or dependability, and confirmability criteria of Guba and Lincoln will be followed to ensure the reliability of the study (35, 36).

## Discussion

Physical exercise stimulates neuroplasticity processes, producing effects on brain structure, function, and connectivity. Exercise causes a change in the metabolic activity of the brain, increasing cerebral blood flow resulting in increased oxygen and glucose metabolism, as well as promoting cardiovascular function and thus reducing peripheral cardiovascular risk factors (e.g., hypertension) for cognitive impairment (37–40). Improved cognitive performance has also been linked to several growth factors whose expression is related to physical exercise, such as insulin-like growth factor 1 (IGF-1), which promotes neuronal growth and improves cognitive performance, and vascular endothelial growth factor (VEGF), which stimulates angiogenesis and vasculogenesis and promotes tolerance to cerebral ischemia (41, 42).

In addition, physical exercise activates BDNF (43), which plays a crucial role in neuroprotection and synaptic plasticity by promoting neurogenesis, cell proliferation, and synaptogenesis in the hippocampus, as well as angiogenesis in other brain areas (44, 45). Finally, exercise contributes to improving memory by elevating dopaminergic activity in the basal ganglia and elevating blood concentrations of other biomarkers (norepinephrine, lactate, etc.) and reduces inflammatory cytokines and oxidative stress, suggesting anti-inflammatory and antioxidant effects in the brain (46, 47).

Considering the benefits of physical exercise for older individuals, it does not seem a good practice to avoid prescribing physical exercise for these individuals. Despite this, physical exercise recommendations have not been fully integrated into primary or geriatric medical practice and are almost absent in the basic training of most health professionals (48). One of the main reasons is the lack of specific tools and recommendations, as even the WHO does not distinguish in its physical activity recommendations between different specific conditions among older people.

Although previous experiences are an important source of evidence, we still lack interventions that are applicable, suitable for all people regardless of gender, and supported by a user-friendly platform. Interventions are needed that include behavior modification models, including active participant learning, intrinsic motivation, self-awareness, and learning over time (49). In addition, these interventions should be culturally adapted and based on individual preferences, accessible to large population groups at low cost, and easily reproducible, including ongoing and personalized support advice. In view of the above, it seems necessary to evaluate the effectiveness of a cognitively demanding cognitive physical exercise home-based eHealth intervention from a population perspective.

This study has some limitations that should be recognized. Although this is a randomized controlled trial, it will not be possible to blind participants to the allocation group, and some bias could be derived from this fact. Second, the measurements and intervention program sessions will be standardized by training the staff, but some variability could not be neglected. Finally, a program based on physical activity will be designed without considering diet interventions, although adherence to Mediterranean diet will be collected, other diet behavior information will not be available.

## Implications

The results of this project aim to impact in the QoL and the well-being of a specific group of population with the objective to cover their needs. The implementations of the findings and their inclusion in guidelines, may directly impact in older people’s life and relatives, through the prevention of morbidity and the reduction of years lost to disability. These benefits may be reflected in the reduction of economic expenditure by reducing the demand for social and health care services.

## Strengths and limitations of this study

Cognitively demanding physical exercise has been proposed to foster cognitive function regardless of age.

This study aims to describe the protocol of a home-based eHealth cognitive-demanding exercise intervention for older adults in cognitive and physical function.

The inclusion of patients in the design of interventions allows them to culturally adapt to individual preferences and improve the participants’ adherence.

The development of the eHealth intervention in two phases and the inclusion of the qualitative approach will ensure the adaptation of the intervention to users’ preferences.

## Ethics statement

The studies involving humans were approved by the Clinical Research Ethics Committee of the ‘Virgen de la Luz’ Hospital in Cuenca approved the study protocol (registration number: 2022/PI3222). The studies were conducted in accordance with the local legislation and institutional requirements. Written informed consent for participation was required from the participants or the participants’ legal guardians/next of kin in accordance with the national legislation and institutional requirements.

## Author contributions

CA-B: Conceptualization, Funding acquisition, Investigation, Methodology, Project administration, Supervision, Writing – original draft. ML-L-T: Investigation, Methodology, Writing – review & editing. AR-H: Conceptualization, Investigation, Methodology, Writing – original draft. IS-D: Investigation, Writing – original draft. LV-S: Conceptualization, Investigation, Methodology, Writing – review & editing. MM-E: Conceptualization, Investigation, Methodology, Writing – review & editing. MV-A: Investigation, Methodology, Validation, Writing – review & editing. BR-M: Conceptualization, Funding acquisition, Investigation, Methodology, Writing – review & editing.

## Glossary

BDNF: brain-derived neurotrophic factor
BMI: Body mass index
cAIx: central augmentation rate
CG: Control Group
COREQ: Consolidated Criteria for Reporting Qualitative Studies
DCCS: Dimension Change Card Sort
ELISA: Enzyme-linked immunosorbent assay
FES-I: Efficacy Scale International
GDS: Geriatric Depression Scale
HRQoL: Health-related Quality of Life
IFCC: International Federation of Clinical Chemistry and Laboratory Medicine
IG: Intervention Group
IGF-1: insulin-like growth factor 1
MEDAS: Mediterranean diet adherence screener
MMSE: Mini-Mental State Examination
NGSP: National Glycohemoglobin Standardization Program
OR: odds ratio
PSQI: Pittsburgh Sleep Quality Index
QoL: Quality of Life
rAIx: radial augmentation rate
SF-12: 12-Item Short Form
SPPB: Short Portable Physical Battery scale
WHO: World Health Organization
6MWT: 6-minute walking test

## References

[1] Amarya S Singh K Sabharwal M. Ageing process and physiological changes In: D’Onofrio G Greco A Sancarlo D, editors. Gerontology. London: IntechOpen (2018)

[2] Bunta AD. It is time for everyone to own the bone. Osteoporos Int. (2011) 22:477–82. doi: 10.1007/s00198-011-1704-0, PMID: 21847769

[3] Faulkner JA Larkin LM Claflin DR Brooks SV. Age-related changes in the structure and function of skeletal muscles. Clin Exp Pharmacol Physiol. (2007) 34:1091–6. doi: 10.1111/j.1440-1681.2007.04752.x 17880359

[4] World Health Organization. Noncommunicable diseases country profiles 2018. Geneva: World Health Organization (2018).

[5] WHO. Dementia: a public health priority. Geneva: World Health Organization (2012).

[6] WHO. First WHO ministerial conference on global action against dementia: meeting report, WHO Headquarters. Geneva, Switzerland: World Health Organization (2015).

[7] Prince M AGM W Ali GC Wu YT Prina M. World Alzheimer report 2015-the global impact of dementia: an analysis of prevalence, incidence, cost and trends. London: Alzheimer’s Disease International (2015).

[8] Wimo A Jönsson L Bond J Prince M Winblad B Alzheimer Disease International. The worldwide economic impact of dementia 2010. Alzheimers Dement. (2013) 9:1–11. doi: 10.1016/j.jalz.2012.11.006 23305821

[9] Winblad B Amouyel P Andrieu S Ballard C Brayne C Brodaty H . Defeating Alzheimer’s disease and other dementias: a priority for European science and society. Lancet Neurol. (2016) 15:455–532. doi: 10.1016/S1474-4422(16)00062-4, PMID: 26987701

[10] Erickson KI Hillman C Stillman CM Ballard RM Bloodgood B Conroy DE . Physical activity, cognition, and brain outcomes: a review of the 2018 physical activity guidelines. Med Sci Sports Exerc. (2019) 51:1242–51. doi: 10.1249/MSS.0000000000001936, PMID: 31095081 PMC6527141

[11] Gavelin HM Dong C Minkov R Bahar-Fuchs A Ellis KA Lautenschlager NT . Combined physical and cognitive training for older adults with and without cognitive impairment: a systematic review and network meta-analysis of randomized controlled trials. Ageing Res Rev. (2021) 66:101232. doi: 10.1016/j.arr.2020.101232, PMID: 33249177

[12] Untari I Subijanto AA Mirawati DK Probandari AN Sanusi R. A combination of cognitive training and physical exercise for elderly with the mild cognitive impairment: a systematic review. J Heal Res. (2019) 33:504–16. doi: 10.1108/JHR-11-2018-0135

[13] Hartwigsen G. Flexible redistribution in cognitive networks. Trends Cogn Sci. (2018) 22:687–98. doi: 10.1016/j.tics.2018.05.008, PMID: 29914734

[14] Schulz KF Altman DG Moher D for the CONSORT Group. CONSORT 2010 statement: updated guidelines for reporting parallel group randomized trials. Ann Intern Med. (2010) 152:726–32. doi: 10.7326/0003-4819-152-11-201006010-00232 20335313

[15] Tong A Sainsbury P Craig J. Consolidated criteria for reporting qualitative research (COREQ): a 32-item checklist for interviews and focus groups. Int J Qual Health Care. (2007) 19:349–357. 17872937 10.1093/intqhc/mzm042

[16] Weintraub S Dikmen SS Heaton RK Tulsky DS Zelazo PD Bauer PJ . Cognition assessment using the NIH toolbox. Neurology. (2013) 80:S54–64. doi: 10.1212/WNL.0b013e3182872ded 23479546 PMC3662346

[17] Folstein MF Robins LN Helzer JE. The mini-mental state examination. Arch Gen Psychiatry. (1983) 40:812. doi: 10.1001/archpsyc.1983.01790060110016 6860082

[18] Boustani M Peterson B Hanson L Harris R Lohr KN. Screening for dementia in primary care: a summary of the evidence for the U.S. preventive services task force. Ann Intern Med. (2003) 138:927–37. doi: 10.7326/0003-4819-138-11-200306030-00015 12779304

[19] Tombaugh TN McIntyre NJ. The mini-mental state examination: a comprehensive review. J Am Geriatr Soc. (1992) 40:922–35. doi: 10.1111/j.1532-5415.1992.tb01992.x 1512391

[20] Ware JE Jr Kosinski M Keller SD. A 12-item short-form health survey: construction of scales and preliminary tests of reliability and validity. Med Care. (1996) 34:220–33. doi: 10.1097/00005650-199603000-00003 8628042

[21] Martínez de la Iglesia J Onís Vilches MC Dueñas Herrero R Albert Colomer C Aguado Taberné C Luque Luque R. Versión española del cuestionario de Yesavage abreviado (GDS) para el despistaje de depresión en mayores de 65 años: adaptación y validación. Medicina de Familia. (2002) 12:620–63. doi: 10.4321/S1131-57682002001000003

[22] Rodríguez DZ Casado MPR Molero SM Jiménez AD Casado TD Labrada GD. Evaluación del cuestionario de Yesavage abreviado versión española en el diagnóstico de depresión en población geriátrica. Revista del Hospital Psiquiátrico de La Habana. (2015) 12. doi: 10.23857/dc.v6i4.1552

[23] ATS Committee on Proficiency Standards for Clinical Pulmonary Function Laboratories. ATS statement: guidelines for the six-minute walk test. Am J Respir Crit Care Med. (2002) 166:111–7. doi: 10.1164/ajrccm.166.1.at1102 12091180

[24] Enright PL McBurnie MA Bittner V Tracy RP McNamara R Arnold A . Cardiovascular health study. The 6-min walk test: a quick measure of functional status in elderly adults. Chest. (2003) 123:387–98. doi: 10.1378/chest.123.2.387 12576356

[25] Guralnik JM Ferrucci L Pieper CF Leveille SG Markides KS Ostir GV . Lower extremity function and subsequent disability: consistency across studies, predictive models, and value of gait speed alone compared with the short physical performance battery. J Gerontol Ser A Biol Med Sci. (2000) 55:M221–31. doi: 10.1093/gerona/55.4.M221 PMC12149745 10811152

[26] Yardley L Beyer N Hauer K Kempen G Piot-Ziegler C Todd C. Development and initial validation of the falls efficacy ScaleInternational (FES-I). Age Ageing. (2005) 34:614–9. doi: 10.1093/ageing/afi196, PMID: 16267188

[27] Martínez-González MA Fernández-Jarne E Serrano-Martínez M Wright M Gomez-Gracia E. Development of a short dietary intake questionnaire for the quantitative estimation of adherence to a cardioprotective Mediterranean diet. Eur J Clin Nutr. (2004) 58:1550–2. doi: 10.1038/sj.ejcn.1602004, PMID: 15162136

[28] Buysse DJ Reynolds CF 3rd Monk TH Berman SR Kupfer DJ. The Pittsburgh sleep quality index: a new instrument for psychiatric practice and research. Psychiatry Res. (1989) 28:193–213. doi: 10.1016/0165-1781(89)90047-4, PMID: 2748771

[29] Charlson ME Carrozzino D Guidi J Patierno C. Charlson comorbidity index: a critical review of clinimetric properties. Psychother Psychosom. (2022) 91:8–35. doi: 10.1159/000521288, PMID: 34991091

[30] Nishiguchi S Yamada M Tanigawa T Sekiyama K Kawagoe T Suzuki M . A 12-week physical and cognitive exercise program can improve cognitive function and neural efficiency in community-dwelling older adults: a randomized controlled trial. J Am Geriatr Soc. (2015) 63:1355–63. doi: 10.1111/jgs.13481 26114906

[31] Giorgi A. The descriptive phenomenological method in psychology: a modified Husserlian approach. Pittsburgh: Duquesne University Press (2009).

[32] Willig C Rogers WS. The SAGE handbook of qualitative research in psychology. London: Sage (2017).

[33] Wilkinson S. Focus Group Research. Qualitative Research Theory, Method and Practice. SAGE Publications, London. (2004); 2: 177–199.

[34] Saunders B Sim J Kingstone T Baker S Waterfield J Bartlam B . Saturation in qualitative research: exploring its conceptualization and operationalization. Qual Quant. (2018) 52:1893–907. doi: 10.1007/s11135-017-0574-8, PMID: 29937585 PMC5993836

[35] Lincoln YS Guba EG. Naturalistic inquiry. Thousand Oaks, CA: Sage (1985).

[36] Mays N Pope C. Assessing quality in qualitative research. BMJ. (2000) 320:50–2. doi: 10.1136/bmj.320.7226.50, PMID: 10617534 PMC1117321

[37] Ainslie PN Cotter JD George KP Lucas S Murrell C Shave R . Elevation in cerebral blood flow velocity with aerobic fitness throughout healthy human ageing. J Physiol. (2008) 586:4005–10. doi: 10.1113/jphysiol.2008.158279, PMID: 18635643 PMC2538930

[38] Ide K Secher NH. Cerebral blood flow and metabolism during exercise. Prog Neurobiol. (2000) 61:397–414. doi: 10.1016/S0301-0082(99)00057-X 10727781

[39] Timinkul A Kato M Omori T Deocaris CC Ito A Kizuka T . Enhancing effect of cerebral blood volume by mild exercise in healthy young men: a near-infrared spectroscopy study. Neurosci Res. (2008) 61:242–8. doi: 10.1016/j.neures.2008.03.012, PMID: 18468709

[40] Van Praag H. Exercise and the brain: something to chew on. Trends Neurosci. (2009) 32:283–90. doi: 10.1016/j.tins.2008.12.007 19349082 PMC2680508

[41] Lin TW Tsai SF Kuo YM. Physical exercise enhances neuroplasticity and delays Alzheimer’s disease. Brain Plast. (2018) 4:95–110. doi: 10.3233/BPL-180073, PMID: 30564549 PMC6296269

[42] Voss MW Soto C Yoo S Sodoma M Vivar C van Praag H. Exercise and hippocampal memory systems. Trends Cogn Sci. (2019) 23:318–33. doi: 10.1016/j.tics.2019.01.006, PMID: 30777641 PMC6422697

[43] Anderson-Hanley C Arciero PJ Nimon J Westen S Okuma N Merz M . Exergaming and older adult cognition: a cluster randomized clinical trial. Am J Prev Med. (2012) 42:109–19. doi: 10.1016/j.amepre.2011.10.016 22261206

[44] Vaynman S Ying Z Gomez-Pinilla F. Hippocampal BDNF mediates the efficacy of exercise on synaptic plasticity and cognition. Eur J Neurosci. (2004) 20:2580–90. doi: 10.1111/j.1460-9568.2004.03720.x, PMID: 15548201

[45] Eriksson PS Perfilieva E Björk-Eriksson T Alborn AM Nordborg C Peterson DA . Neurogenesis in the adult human hippocampus. Nat Med. (1998) 4:1313–7. doi: 10.1038/3305 9809557

[46] Liang J Wang H Zeng Y Qu Y Liu Q Zhao F . Physical exercise promotes brain remodelling by regulating epigenetics, neuroplasticity and neurotrophins. Rev Neurosci. (2021) 32:615–29. doi: 10.1515/revneuro-2020-0099, PMID: 33583156

[47] Cotman CW Berchtold NC Christie LA. Exercise builds brain health: key roles of growth factor cascades and inflammation. Trends Neurosci. (2007) 30:464–72. doi: 10.1016/j.tins.2007.06.011, PMID: 17765329

[48] Izquierdo M Duque G Morley JE. Physical activity guidelines for older people: knowledge gaps and future directions. Lancet Heal Longev. (2021) 2:e380–3. doi: 10.1016/S2666-7568(21)00079-9, PMID: 36098146

[49] Martinho D Carneiro J Corchado JM Marreiros G. A systematic review of gamification techniques applied to elderly care. Artif Intell Rev. (2020) 53:4863–901. doi: 10.1007/s10462-020-09809-6

